# Role of vanadium ions substitution on spinel MnCo_2_O_4_ towards enhanced electrocatalytic activity for hydrogen generation

**DOI:** 10.1038/s41598-023-29081-2

**Published:** 2023-02-06

**Authors:** M. J. S. Mohamed, Y. Slimani, M. A. Gondal, M. A. Almessiere, A. Baykal, M. Hassan, A. Z. Khan, Anurag Roy

**Affiliations:** 1 Laser Research Group, Physics Department, IRC-Hydrogen and Energy Storage, King Fahd University of Petroleum and Minerals (KFUPM), Dhahran, 31261 Saudi Arabia; 2 Department of Biophysics, Institute for Research and Medical Consultations (IRMC), Imam Abdulrahman Bin Faisal University, P.O. Box 1982, Dammam, 31441 Saudi Arabia; 3 K.A. CARE Energy Research and Innovation Center, King Fahd University of Petroleum and Minerals, Dhahran, 31261 Saudi Arabia; 4 Department of Physics, College of Science, Imam Abdulrahman Bin Faisal University, P.O. Box 1982, Dammam, 31441 Saudi Arabia; 5 Department of Nanomedicine Research, Institute for Research and Medical Consultations (IRMC), Imam Abdulrahman Bin Faisal University, P.O. Box 1982, Dammam, 31441 Saudi Arabia; 6 School of Physics, Minhaj University Lahore, Punjab 54770, Pakistan; 7 Department of Chemistry, Forman Christian College, Lahore, 54600 Pakistan; 8 Solar Energy Research Group, Environment and Sustainability Institute, Faculty of Environment, Science and Economy, University of Exeter, Cornwall, TR10 9FE UK

**Keywords:** Chemistry, Engineering, Materials science, Nanoscience and technology

## Abstract

Improving efficient electrocatalysts (ECs) for hydrogen generation through water splitting is of significant interest in tackling the upcoming energy crisis. Sustainable hydrogen generation is the primary prerequisite to realizing the future hydrogen economy. This work examines the electrocatalytic activity of hydrothermally prepared vanadium doped MnCo spinel oxide microspheres (MC), MnV_x_Co_2−x_O_4_ (V_x_-MnCo MC, where x ≤ 0.4) in the HER (hydrogen evolution reaction) process. Magnetization measurements demonstrated a paramagnetic (at high temperatures) to a ferrimagnetic (at low temperatures) transition below the Curie temperature (Tc) in all the samples. The magnetization is found to intensify with the rising vanadium content of MCs. The optimized catalyst V_x_-MnCo MCs (x = 0.3) outperformed other prepared ECs with a Tafel slope of 84 mV/dec, a low onset potential of 78.9 mV, and a low overpotential of 85.9 mV at a current density of 10 mA/cm^2^, respectively. The significantly improved HER performance of hydrothermally synthesized V_x_-MnCo MCs (x = 0.3) is principally attributable to many exposed active sites, accelerated electron transport at the EC/electrolyte interface, and remarkable electron spectroscopy for chemical analysis (ECSA) value was found ~ 11.4 cm^2^. Moreover, the V_x_-MnCo MCs (x = 0.3) electrode exhibited outstanding electrocatalytic stability after exposure to 1000 cyclic voltametric cycles and 36 h of chronoamperometric testing. Our results suggest a feasible route for developing earth-abundant transition metal oxide-based EC as a superior electrode for future water electrolysis applications.

## Introduction

Hydrogen is one of the most sustainable, and low-cost technologies for large-scale clean energy production to solve the global energy problem^1^. Fossil fuels consume a significant amount of natural resources and produce undesired products like CO_2_, which creates an alarming situation due to the greenhouse effect^2^. Using hydrogen and oxygen as fuel is considered an excellent candidate source of clean energy to meet the growing energy demands, climate change, etc^3^. The electro-catalytic water-splitting demonstrates a sustained energy source for generating hydrogen/oxygen at a large scale attributable to high purity, high efficiency and zero pollutant^4,5^. Several alloys and materials oxides are well-known candidates for developing hydrogen/oxygen electrodes. As a result, certain oxides with transition metals, such as spinel structures, have high electronic conductivity and exhibit significant electrochemical activity in the hydrogen/oxygen evolution reaction (HER/OER) because of their outstanding chemical and physical characteristics. A selection of parameters such as temperature, preparation method, substitution ions and pH of the precursor solution substantially improved the catalytic activity of spinels^6–8^. Accordingly, adjusting the spinel oxide samples' phase can optimise the hydrogen evolution functioning of the sample^9–12^.

MnCo_2_O_4_ has an inverse spinel structure in which Mn^2+^ ions and the Co^2+^ ions have occupied the octahedral (Oh, B) sites and are evenly distributed over the Oh and tetrahedral (Td, A) sites. The cation substitution into MnCo_2_O_4_ spinel can significantly alter its electrical and magnetic features because the electron transfer distance between B and B is short, which enhances the electron transfer and the electrical conductivity^13,14^. The effect of cation composition on the magnetic features of Co_2−x_Bi_x_MnO_4_ (0.0 ≤ x ≤ 0.3) mixed cubic spinel system was examined by Rajeevan et al^15^. The Ni^2+^ substitution on magnetic properties of MnCo_2_O_4_ was also studied by Wang et al^16^. The literature survey showed that the influence of cation substitution on the magnetic properties of MnCo_2_O_4_ is still low and needs more investigation.

Newly, numerous research has been dedicated to developing electrocatalysts for electrochemical Hydrogen Evolution Reaction (HER) utilizing nanomaterials. EWS (electrochemical water splitting) is a favorable methodology for hydrogen production^17,18^. To promote the development of hydrogen gas as fuel, a suitable catalyst with low-cost, natural abundance, excellent electrochemical activity, ease of preparation, and long-term stability for both HER^19,20^ and OER (oxygen evolution reaction) will be employed be employed be critical for electrochemical water splitting^21^.

Spinel oxides are emerging as a promising electrocatalyst class for EWS, including MnCo_2_O_4_^22^, NiCo_2_O_4_^23^, Co_3_O_4_^24^, etc. The cobalt-based spinel oxides (MnCo_2_O_4_, NiCo_2_O_4_, ZnCo_2_O_4_, CuCo_2_O_4_) have demonstrated significant performance in electrocatalytic OER^25^. Moreover, MnCo_2_O_4_ has also been investigated as a potential applicant for energy storage applications, including batteries and supercapacitors, in terms of large capacitance, long cycling stability, and high operating voltage. These superior electrochemical properties are attributed to the multiple oxidation states (+2, +3, +4) of Mn and Co in magnetic spinel oxides^26^. Different types of spinel oxides (Co_3_O_4_, ZnCo_2_O_4_, MgV_2_O_4_, NiCo_2_O_4_, CuCo_2_O_4_, and NiMn_2_O_4_ have been explored as battery-type electrode materials in recent years due to multiple valence states and rich redox reactions^27^. Yan et al.^28^ synthesized the vanadium-doped nickel sulfide nanoflower via a hydrothermal approach for a high-performance supercapacitor. They observed that the increase in surface area and interfacial position of electrode materials promoted the rapid transport of electrolyte ions and electrons and improved the electrochemical performance after vanadium doping. The eco-friendly hydrogen generation by urea-assisted water electrolysis using spinel M_2_GeO_4_ (M = Fe, Co) as an active electrocatalyst was done by the Choi research group^29^. Alqarni et al.^30^ studied the synthesis and high current supercapacitor applications of vanadium intercalated spinel ferrite (Co_0.5_Ni_0.5_V_x_Fe_1.6− x_O_4_) electrodes, and results showed a facile and low-cost production of vanadium doped ternary ferrite nanomaterials illustrated excellent high-rate electrochemical performance.

The present study demonstrated a novel V-doped MnCo microsphere spinel oxide (MC) synthesised via a hydrothermal route and investigated the physical properties and electrochemical features. The V doped MnCo microspheres spinel oxide was coated with glassy carbon electrodes to be used as cathodes to explore their performance for HER in an acidic medium. Vanadium has a variety of stable oxidation states (+2, +3, +4, and +5). Its high oxidation states (+4 and +5) can store charge in a positive potential range, thus supplying a favourable pseudo capacitance^28,31^. Boosting the specific capacitance of the electrode dramatically, ions with two (or more) ions of different valence states have higher charge storage capacity and more abundant redox reactions than most other transition metal ions29.

## Materials and methods

### Synthesis of Mn spinel oxide microspheres (V_x_-MnCo MCs)

The one-step hydrothermal process manufactured the V_x_-MnCo (x ≤ 0.4) MCs. Stoichiometric amount of Co(NO_3_)_2_⋅6H_2_O, Mn(NO_3_)_2_⋅6H_2_O, VCl_3_, and urea (CH_4_N_2_O) were used as initial materials. The stoichiometric quantities of metal salts were thawed in 20 ml DI H_2_O by stirring at room temperature (RT), and then 1.2 g of urea (CH_4_N_2_O) were thawed in 30 ml DI water. Both mixtures were stirred and sonicated for 30 min. The solution was installed into an autoclave (stainless steel) and heated for 12 h at 180 °C. Finally, the solid product was washed with warm deionised water, filtered, and dried in an oven.

For the working electrode preparation, 80 µL of 5% Nafion solution and 4 mg of catalyst were dissolved in a mixture of de-ionized water and ethanol in 1 mL (4:1 in volume ratio). The ultrasonication was utilised for 30 min. to guarantee that the solution was fully dispersed. Then, 5 μL catalyst ink solution was deposited on the polished GC electrode and dried at 80 °C for 2 h to achieve the catalyst loading of 0.285 mg/cm^2^.

The crystal structure of the synthesized MCs was analyzed via a Rigaku Benchtop Miniflex XRD (X-ray diffractometer). The surface morphology and chemical composition were implemented with SEM (scanning electron microscopy) and FEI with the model Titan ST model HR-TEM, TEM (transmission and high-resolution electron microscopies) microscopes along with EDX (energy dispersive X-ray spectroscopy).

### Electrochemical characterization

The Linear sweep voltammetry (LSV), Cyclic voltammetry (CV), Chronopotentiometry (CP), and Electrochemical impedance spectroscopy (EIS) were performed in a 0.5 M H_2_SO_4_ solution with a typical three-electrode system using an AutoLab PGSTAT302N electrochemical workstation. Glassy carbon (GC) electrodes coated with V-MnCo MCs were used as working electrodes, a saturated Ag/AgCl electrode was employed as the reference electrode, and the Pt wire was utilized as the counter electrode. Nernst equation was used to calibrate all the potentials to E_RHE_ (Reversible hydrogen electrode potential) was used to calibrate all the potentials for HER measurements using Eq. (1)^1^.

1 $${\mathrm{ERHE }=\mathrm{ Eappl }+ 0.059\mathrm{ pH }+\mathrm{ E}}_{\mathrm{Ag}/\mathrm{AgCl}}^{\mathrm{o}}$$

where ${\mathrm{E}}_{\mathrm{Ag}/\mathrm{AgCl}}^{\mathrm{o}}$ is standard electrode potential equivalent to 0.198 V at 25 °C. The scan rate for LSV measurements was 5 mV s^−1^. The electrochemical chronopotentiometry was used to measure the stability of the V-MnCo MCs at a fixed current of −10 mA/cm^2^. EIS measurements were measured by the same potentiostat (PGSTAT302N Autolab) equipped with a frequency module analyzer (FRA2).

## Results & discussion

### Phase and morphology analysis

**Figure 1:**
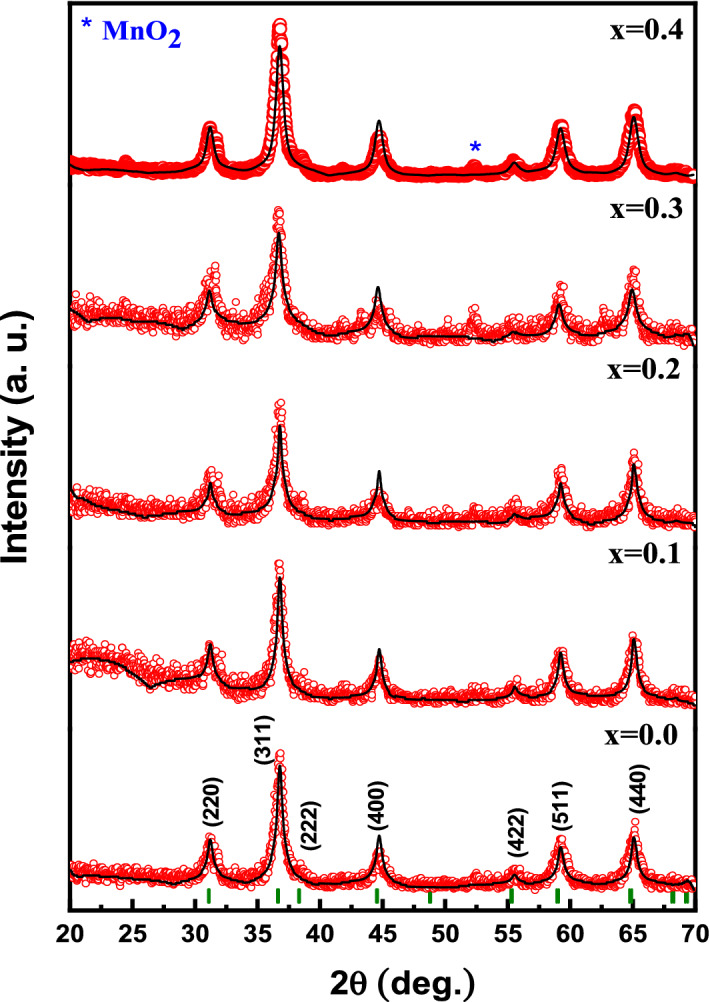
XRD powder patterns of V_x_-MnCo (x ≤ 0.4) MCs.

The phase purity analysis of V_x_-MnCo MCs was investigated through powdered XRD analysis and refined by Match! software and full proof, as depicted in Fig. 1. The XRD pattern disclosed the prominent dominant peaks of cubic spinel oxide structure with relatively low-intensity peaks associated with the trace of MnO_2_. All diffraction peaks in Fig. 1 matched well with the standard international centre for diffraction data (file no. 23-1237) of MnCo_2_O_4_. Rietveld analysis of experimental XRD pattern data revealed the lattice parameters, cell volume, reliability factors and crystallite size, as seen in Table 1. The lattice parameters increased with increasing the 'x' due to the expansion in the lattice due to a mismatch in ionic radii. The crystallite sizes were calculated between 16.0 and 22.4 nm implementing Scherrer’s formula for the most intense peak (311)^32^.

**Table 1:** Refined cell parameters of V_x_-MnCo (x ≤ 0.4) MCs.

*x*	*a* = *b* = *c* (Å)	*V* (Å^3^)	*D*_*XRD*_ (nm) ± 0.10	*χ*^2^ (*chi*^2^)	*R*_*Bragg*_
0.0	8.1041	532.25	19.3	1.2	21.1
0.1	8.1051	532.43	21.1	0.8	27.1
0.2	8.1183	535.05	22.4	0.5	29.5
0.3	8.1192	535.22	17.2	1.3	30.3
0.4	8.1278	536.92	16.0	1.3	29.3

**Figure 2:**
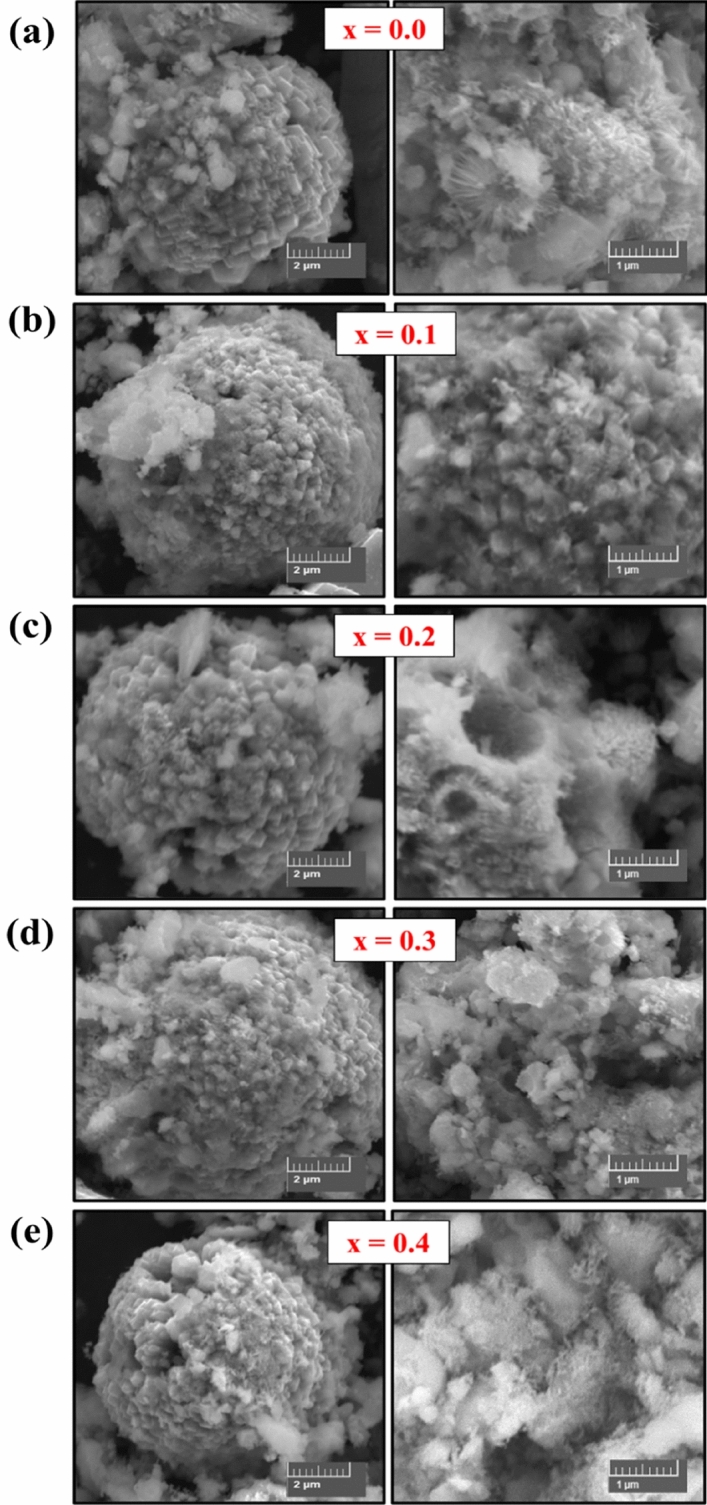
SEM microstructural images of V_x_-MnCo samples for **(a)** without V doping followed by x = **(b)** 0.1, **(c)** 0.2, **(d)** 0.3, and **(e)** 0.4 at different magnifications.

**Figure 3:**
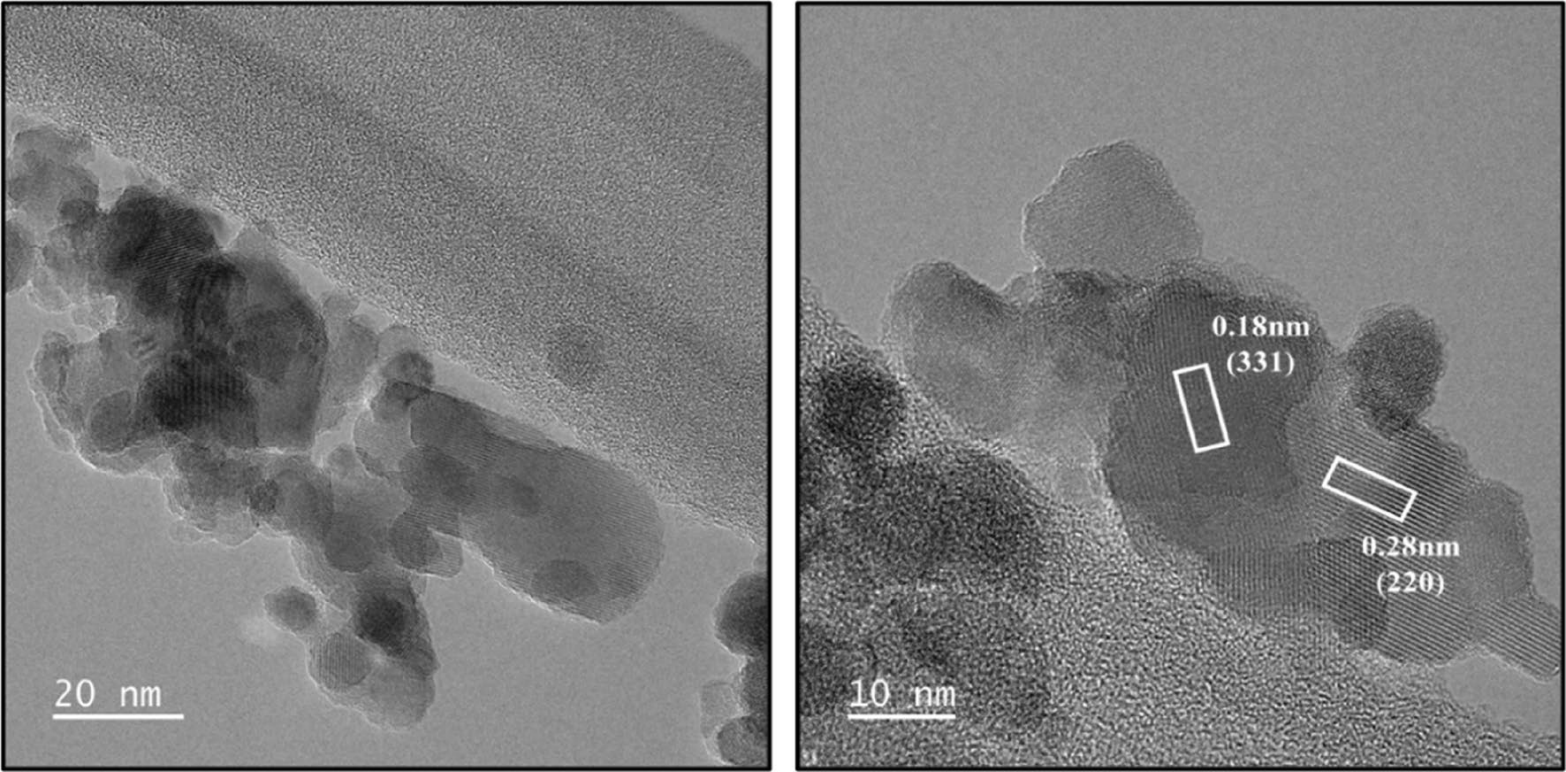
TEM (left) and HR-TEM (right) micrographs of V_x_-MnCo (x = 0.3) MCs.

**Figure 4:**
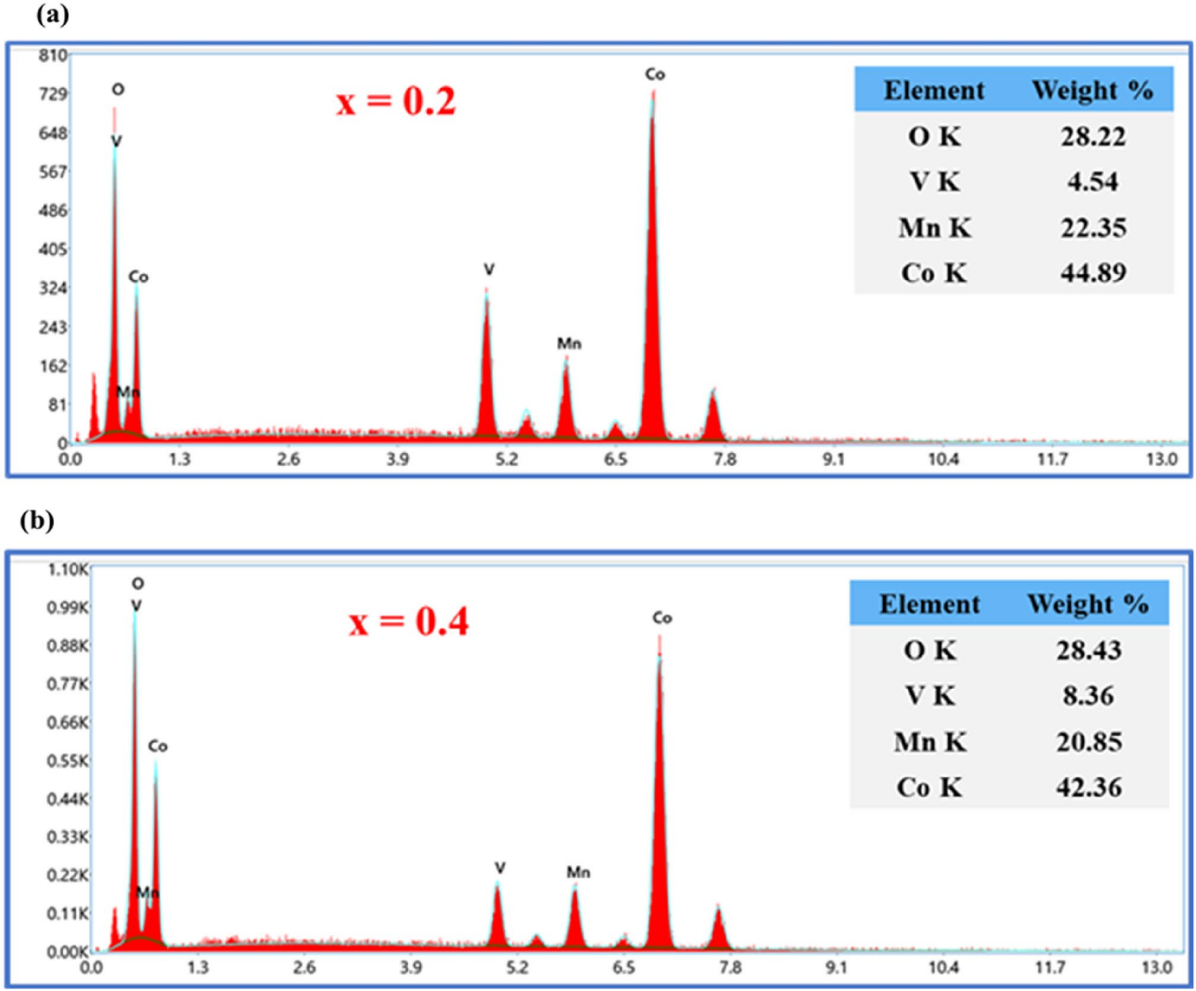
EDX spectrum of V_x_-MnCo samples for x = **(a)** 0.2 MCs, and **(b)** 0.4 MCs.

The surface morphology and shape of V_x_-MnCo MCs were investigated by SEM analysis with different magnifications, as shown in Fig. 2. The images exhibited the different sizes of spherical particles. The high magnification images revealed that the spherical particles consisted of highly agglomerated small cubic particles, giving rise to the spherical particles with a rough surface. However, an enormous aggregation of the particles was dominated for the V_x_-MnCo MCs (x = 0.3) after the stability test, as depicted in Fig. 12b. To further confirm the morphology and structure of V_x_-MnCo MCs (x = 0.3), TEM and HR-TEM were employed, as clear from Fig. 3 shows the assembling of spherical particles, and HR-TEM approved the spinel phase of the synthesized samples. The well-defined D-spacing of 0.28 and 0.18 nm are assigned to the (220) and (331) facets of V_x_-MnCo MCs following the XRD results. The presence of Co, Mn, V and O elements in V_x_-MnCo (x = 0.2 and 0.4) MCs was confirmed by the EDX analysis as illustrated in Fig. 4a,b. Moreover, the V content was increased, and Mn and Co concentration was suppressed in the EDX spectra from x = 0.2 and 0.4, which indicates that V was successfully doped into MnCo MCs and consistent with XRD and TEM analysis.

### X-ray photoelectron spectroscopy analysis

**Figure 5:**
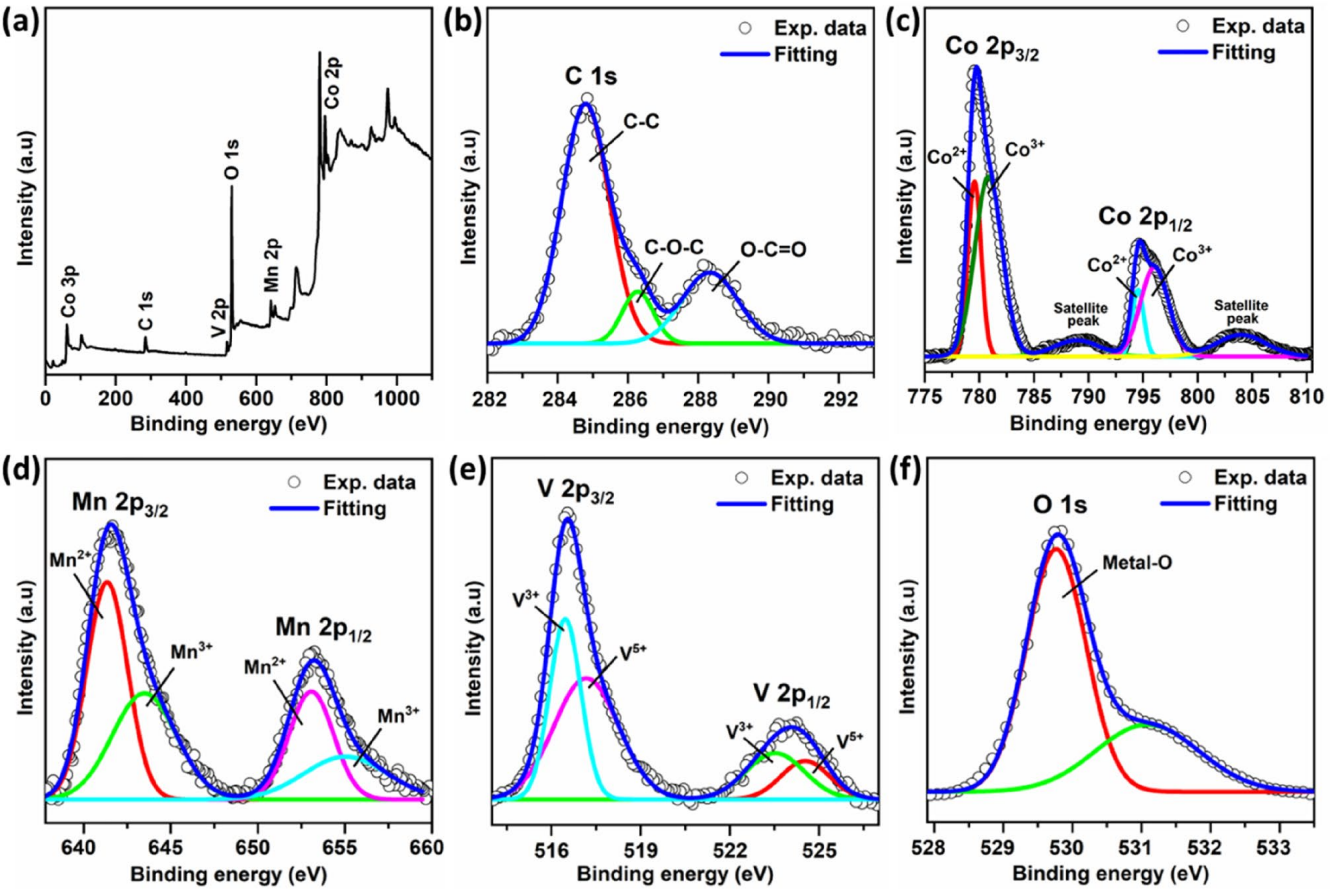
XPS analysis of V_x_-MnCo MCs (x = 0.3) sample represents **(a)** survey scan and core level spectrum of **(b)** C 1s, **(c)** Co 2p, **(d)** Mn 2p, **(e)** V 2p, **(f)** O 1s.

The chemical composition and oxidation state of V_x_-MnCo MCs (x = 0.3) magnetic nanoparticles were determined using XPS analysis. A comprehensive survey scan was conducted to identify the sample elements as presented in Fig. 5a. In addition, the deconvoluted characteristic peaks of each element were shown separately in Figs. 5b–f. The core level spectrum of the C 1s state was observed at 284.8 eV, further deconvoluted into three characteristic peaks, as shown in Fig. 5b. The peaks observed at binding energies of 284.8 eV, 286.3 eV, and 288.3 eV are assigned to C–C, C–O–C and O–C=O bonds, respectively. Figure 5c shows the core level peaks of Co at a binding energy of 779.7 eV and 794.7 eV, which correspond to Co 2p_3/2_ and Co 2p_1/2,_ respectively^33^. The deconvolution of the core peak of Co 2p_3/2_ introduced two characteristic peaks at 779.5 eV and 780.9 eV corresponding to Co^2+^ and Co^3+^ states, respectively.

Similarly, Co 2p_1/2_ spectrum was deconvoluted into two peaks at 794.5 eV and 795.9 eV indicating the presence of Co atoms at two different geometrical positions in the spinal structure. In addition, two satellite peaks of Co 2p_3/2_ and Co 2p_1/2_ spectra were also observed at 789.0 eV and 804.0 eV, respectively. Mn 2p_3/2_ and Mn 2p_1/2_ core spectra were observed at 641.6 eV and 653.2 eV, respectively^34^ as presented in Fig. 5d. As a result of deconvolution, the core spectra of Mn were split into characteristic peaks associated with Mn^2+^, and Mn^3+^ states, respectively^35^. The spin–orbit doublet of core level spectra of V 2p_3/2_ and V 2p_1/2_ were detected at 516.5 eV and 524.1 eV, respectively^36^, as shown in Fig. 5e. The further deconvolution of the V 2p core spectrum introduces two peaks in each case, indicating the presence of V^3+^ and V^5+^ ionic states. Figure 5f represents the deconvoluted spectrum of O 1 s species exhibiting characteristic peaks at 529.7 eV and 531.1 eV, respectively^37^. The high-intensity peak at 529.7 eV represents the metal oxide bonding, whereas the low-intensity broad peak centered around 531.2 eV is associated with the adsorbed hydroxyl groups and unreacted carbonates.

### Magnetic properties

**Figure 6:**
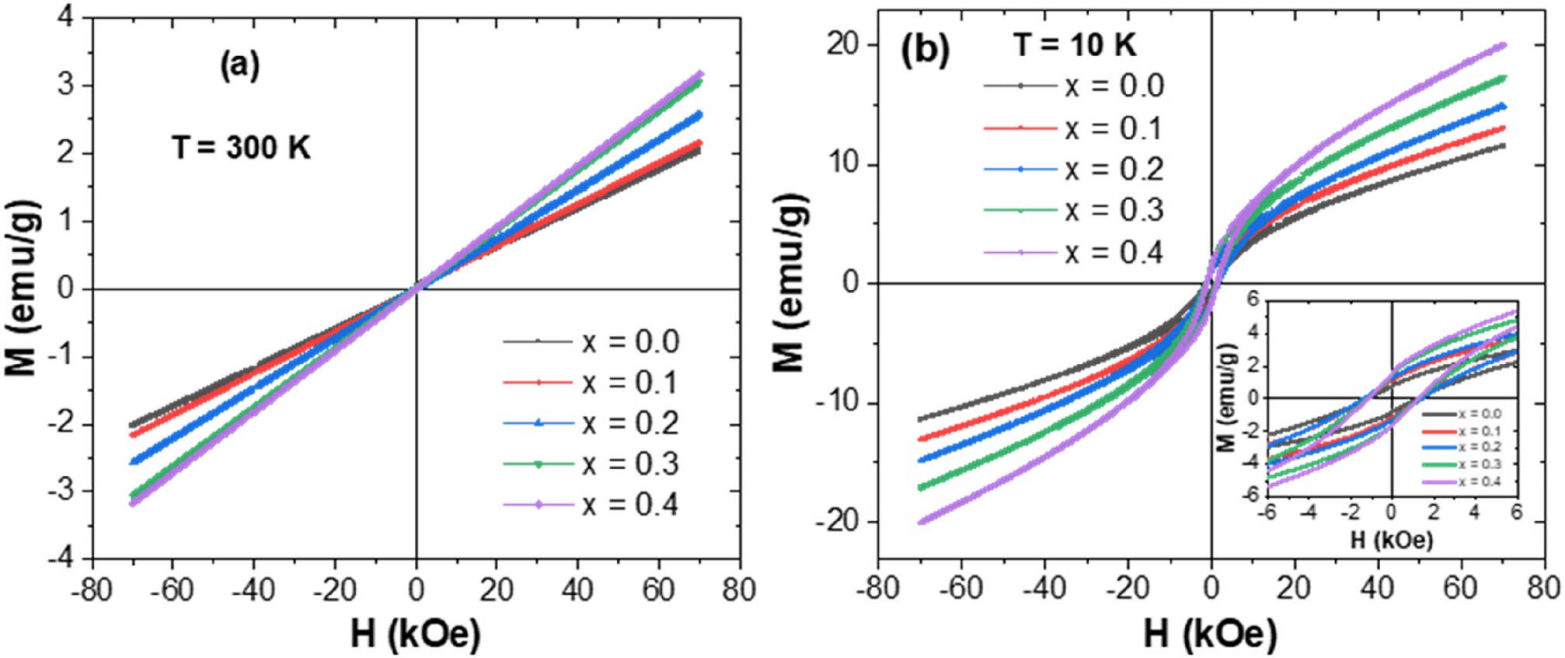
Magnetic hysteresis loops of V_x_-MnCo (x ≤ 0.4) MCs recorded at **(a)** T = 300 K and **(b)** T = 10 K.

Figure 6a,b present the variations of magnetization against the applied magnetic field (M-H) for different V_x_-MnCo (x ≤ 0.4) MCs registered under H = ± 70 kOe and at temperatures of 300 and 10 K, respectively. M-H results measured at room temperature showed almost linear curves for all samples, indicating their paramagnetic behaviors at 300 K. an apparent hysteresis loop-like feature could be observed in the M-H curves at low magnetic fields measured at 10 K as pointed out by the enlarged view in the inset of Fig. 6b. The opened hysteresis loops at 10 K for all prepared samples reflect the transformation to a ferrimagnetic state at lower temperatures. In spinel oxides, three main magnetic interactions exist among the tetrahedral (A) and octahedral (B) sites via the intermediate O^2−^ ions through superexchange interactions. For instance, the weak superexchange interactions Co^2+^–O^2–^ Co^3+^–O^2^– Co^2+^ maintained an antiferromagnetism effect in Co_3_O_4_ composition^38^. Substituting some Co^3+^ ions with Mn^3+^ ions to form a MnCo_2_O_4_ sample will significantly affect the magnetic exchange interactions. Indeed, the inclusion of Mn^3+^ ions having a larger size than Co^3+^ ions will distort the oxygen octahedron and hence frustrate the tetrahedral site, provoking an insufficient magnetic ordering in the MnCo_2_O_4_ (*x* = 0.0 in this case) MCs. Accordingly, the manifestation of ferrimagnetism in MnCo_2_O_4_ MCs at lower temperatures could be ascribed to the canting effect of antiferromagnetically ordered spins via structural distortions and breakdown of the equilibrium among the Co^2+^ sublattices’ antiparallel magnetization caused by the inclusion of Mn^3+^ ions^39^.

Catalysis and magnetism may be different manifestations of some more fundamental atomic properties. But the current chief applications of magnetism to catalysis are in structural studies of catalytic solids.

**Figure 7:**
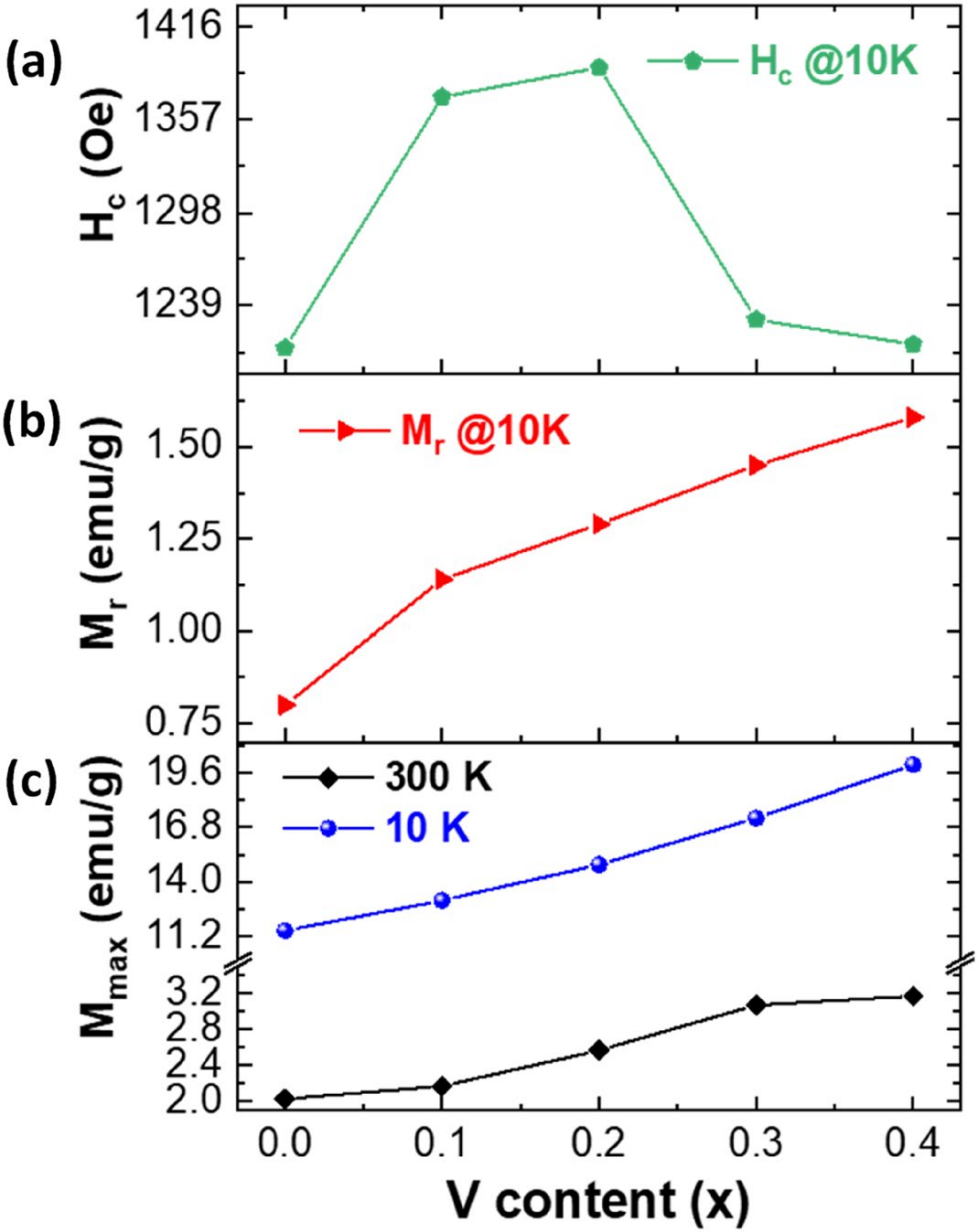
Evolutions in **(a)** M_max_ (± 70 kOe) at 300 and 10 K, **(b)** M_r_ at 10 K, and **(c)** H_c_ at 10 K concerning “x”.

On the other hand, the variation in magnetic parameters is probably a result of the inclusion of vanadium ions that will cause inhomogeneity and intrinsic pinning of the domain walls. Due to the dissimilarity in ionic radii of vanadium and cobalt ions, a change in the exchange interactions could be caused by the local crystal fields. Such an effect may conduce to the generation of energy barriers that could considerably affect the magnetization reversal process at low temperatures.At low temperatures (10 K), the vanadium composition dependence of the magnetic parameters like magnetization M_max_ achieved at 70 kOe, remanent magnetization (M_r_), and coercive field (H_c_) is presented in Fig. 7. The room temperature (300 K) magnetization M_max_ achieved at 70 kOe was also presented in Fig. 7a. There is a continuous increase in the magnetization M_max_ with increasing vanadium content in comparison to the parent sample at both 300 and 10 K. For different prepared compositions, M_max_ (at 70 kOe) increased as the temperature decreased from 300 K down to 10 K. For example, M_max_ (at 70 kOe) is equal to 2.0 and 3.2 emu/g at 300 K for samples with x = 0.0 and 0.4, respectively, and increased to about 11.5 and 20.0 emu/g at 10 K. As the temperature decreases, M_max_ (at 70 kOe) increases since the thermal fluctuations contributing to demagnetization with be lowered at lower temperatures. Hence, M_max_ (at 70 kOe) and M_r_ and H_c_ will rise. In other words, the thermal agitation will tend to decrease as the temperature decreases. Hence the easy magnetization rotation would be more dominant than the thermal agitations, leading to an increase in global magnetization magnitude. As the composition content (x) rises, it is found that M_max_ (at 70 kOe) and M_r_ values increase simultaneously at low temperatures (T = 10 K). For example, M_max_ (at ± 70 kOe) is about 2.3 emu/g (at 300 K) and 11.5 emu/g (at 10 K) for the non-doped sample (*x* = 0.0) and attained about 3.2 emu/g (at 300 K) and 20.0 emu/g (at 10 K) for the product with *x* = 0.4. M_r_ measured at 10 K is about 0.8 emu/g for the non-doped sample (x = 0.0) and attained about 1.58 emu/g for the product with x = 0.4. For the intermediate compositions, M_r_ values are about 13.1, 14.9, and 17.3 emu/g for samples with x = 0.1, 0.2, and 0.3, as shown in Fig. 7b, respectively.

The magnetization values continuously increase with vanadium substitution. However, the variation in H_c_ value showed an anomaly at x > 0.2. The H_c_ increases first with increasing vanadium content (x) up to 0.2 but then drops with additional rising vanadium content (x > 0.2), as shown in Fig. 7c. It is well-known that coercivity H_c_ dramatically depends on the crystallite size^40,41^. According to XRD results, it is found that the crystallite size increases with increasing *x* content up to 0.2 but falls with further increasing *x* content. At 10 K, H_c_ is about 1212.1 Oe for the non-doped sample (x = 0.0), which progressively increases up to 1371.6 and 1390.3 Oe for the products with x = 0.1 and 0.2, respectively, and starts to slightly decrease with the further increase in vanadium content (H_c_ = 1230.2 Oe for x = 0.3 and H_c_ = 1214.5 Oe for x = 0.4). The registered H_c_ values are non-negligible, indicating the complex magnetic features of the present samples at low temperatures. The variations in M_max_ (at 70 kOe) could be well-explained by the distribution of cations in different sites and hence, the variation in the net magnetic moment of the whole system.

For non-substituted MnCo_2_O_4_ MCs, the cations are distributed among Td and Oh sites as follows: (Co^3+^)_A_[Co^2+^Mn^3+^]_B_O_4_. The Td sites are occupied by Co^3+^ ions (spin S = 2 and g = 2) with a magnetic moment of about$;close\mu \left(A\right)=4.9{\mu }_{B}$. On the other side, the Oh sites are occupied by Co^2+^ and Mn^3+^ ions with high spin state S = 3/2 and 2, respectively. Hence, the magnetic moment of B site is equal to the ${\mu \left(B\right)=\sqrt{{\left({\mu }_{{Co}^{2+}}\right)}^{2}+{\left({\mu }_{{Mn}^{2+}}\right)}^{2}}=\sqrt{{\left(3.87\right)}^{2}+{\left(4.9\right)}^{2}}=6.24\mu }_{B}$. Accordingly, it could be noticed that the orbital angular momentum of the magnetic ions situated at B sites is completely quenched. Upon vanadium doping, the cations are distributed as ${\left({Co}^{3+}\right)}_{A}{\left[{Co}_{2-x}^{2+}{V}_{x}^{2+}{Mn}^{3+}\right]}_{B}{O}_{4}$ where vanadium ions occupy Oh sites once they substitute some ions of Co^2+^. In this configuration, the three ions residing in the B site display magnetic moments of $\mu \left({Co}^{2+}\right)=3.87{\mu }_{B}$, $\mu \left({V}^{2+}\right)=1.73{\mu }_{B}$, and $\mu \left({Mn}^{2+}\right)=4.9{\mu }_{B}$. The substitution of some magnetic Co^2+^ ions residing in B sites by some V^2+^ ions with lower magnetic moments will reduce the magnetic moment of B sites. Theoretically, the net magnetic moment of the whole system will decrease with increasing *x* content since the magnetic moment of A sites is constant. However, this is different in the present study, where an increment in magnetization with vanadium substitution has been observed. This indicates that other factors govern the magnetic behavior of present samples. Accordingly, further investigations will be done by our research group as further study.

**Figure 8:**
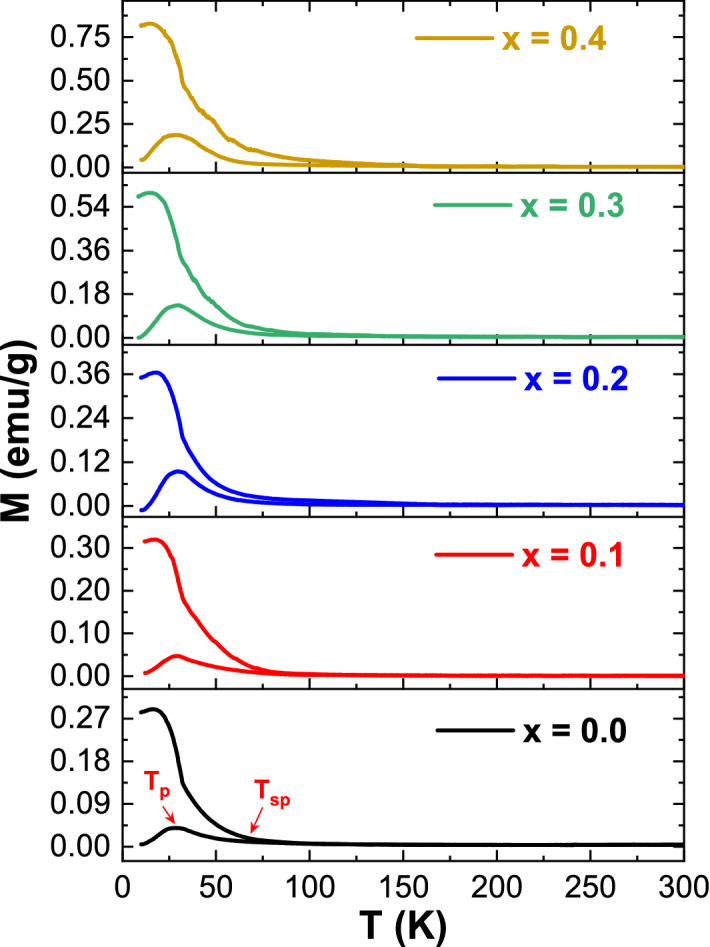
M–T graph (temperature dependence of magnetization) of V_x_-MnCo (x ≤ 0.4) MCs measured under FC and ZFC conditions and a dc-magnetic field of 100 Oe.

Figure 8 displays the temperature (10 K ≤ T ≤ 325 K) dependence of magnetization (M–T) for different V_x_-MnCo (x ≤ 0.4) MCs carried out under FC and ZFC conditions. A dc-magnetic field of about H_app_ = 100 Oe has been applied. Hereafter, M–T curves measured under ZFC, and FC conditions were noted as M_ZFC_(T) and M_FC_(T) curves, respectively. The general shape of M_ZFC_(T) curves is practically comparable for different products. The marked transformation in the slope of both M_ZFC_(T) and M_FC_(T) plots indicates the well-known ferrimagnetic Curie temperature T_c_. These magnetic transitions at T_c_ emphasize the ferrimagnetic behavior of the prepared products. For V_x_-MnCo (x ≤ 0.4) MCs, Tc values are lesser than those registered for bulk MnCo_2_O_4_ MCs (T_c_ ~ 185 K), which is mainly attributed to the effects of finite size^42^. The V_x_-MnCo (x ≤ 0.4) MCs display a distinctive separation between the M_ZFC_(T) and M_FC_(T) curves at a certain temperature noted as T_sp_ < T_c_ (Fig. 8). M_FC_(T) plot declines progressively with the rising temperature and merges more than M_ZFC_(T) plot. The magnetization’ thermal irreversibility is usually detected under Curie temperature. This is because of the existence of high magneto-crystalline anisotropy in prepared samples. The observed irreversibility is comparable to the previous findings for MnCo_2_O_4_^43^ and certain ferrimagnetic and ferromagnetic oxides.

Furthermore, all products showed maximum magnetizations peak at a temperature noted as T_p_ (lower than the Curie temperature) in the M_ZFC_(T) curves, and afterwards, it falls to shallow values. Typically, the appearance of a peak around T_P_ could be ascribed to either the spin-glass state or the blocking phenomenon caused by the finite sizes and surficial effects in—V_x_-MnCo (x ≤ 0.4) MCs. Moreover, the presence of such a peak could be explained based on the Hopkinson effect, a competing effect between the applied magnetic field and the magneto-crystalline anisotropy that varies with the temperature. Like what was reported in the above M-H part, the impact of domain wall pinning could also influence the shape of M_ZFC_(T) curves. Indeed, by heating the specimen with a low magnetic field application, the domain walls’ mobility could be raised. This means that the domain wall pinning effect will be diminished with the increase in temperature. Hence the walls could be moved easily toward the direction of the applied magnetic field, which will provoke a small increment in the magnetization.

Above Tc, the product will be demagnetized; hence, the magnetization drops to almost zero at Tc. On the other hand, the thermal agitation will tend to increase as the temperature increases, leading to a reduction in magnetization. Nevertheless, at T < Tc, the rotation of easy magnetization could be more dominant than the thermal agitations, leading to a global rise in magnetization.

**Figure 9:**
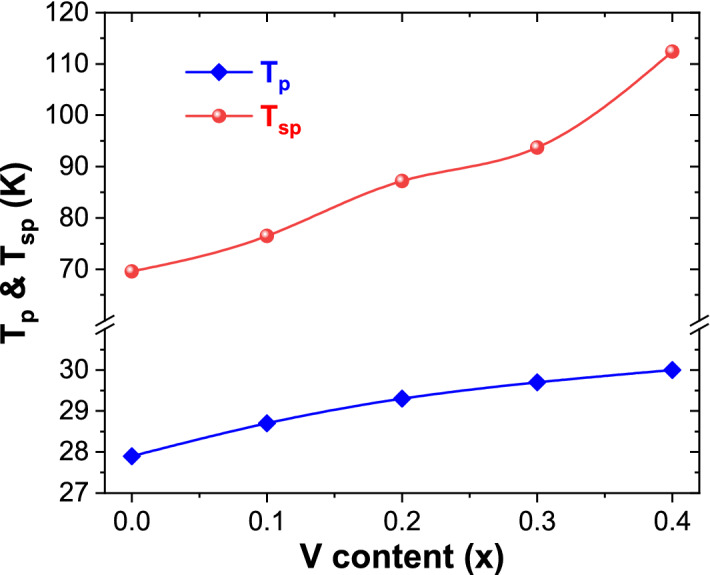
Variations in T_p_ and T_sp_ temperatures versus vanadium content.

Consequently, a maximum peak is detected in the M_ZFC_(T) curves below T_c_^44^. Furthermore, it was evident that T_c_ shifts progressively towards higher temperatures with increasing “x”. This could be correlated with the slight increment of crystallites/grains size as the vanadium content increases^45^. The variations in the critical temperatures T_sp_ and T_p_ as a function of vanadium content are shown in Fig. 9. It is noticed that T_p_ and T_sp_ shift gradually towards higher temperatures with increasing “x”. For instance, T_p_ is about 27.9 K for non-doped MCs, which has enhanced steadily from 28.7, 29.3, 29.7, to 30.0 K for x = 0.1, 0.2, 0.3, and 0.4 compositions, respectively.

### Hydrogen evolution reaction studies

**Figure 10:**
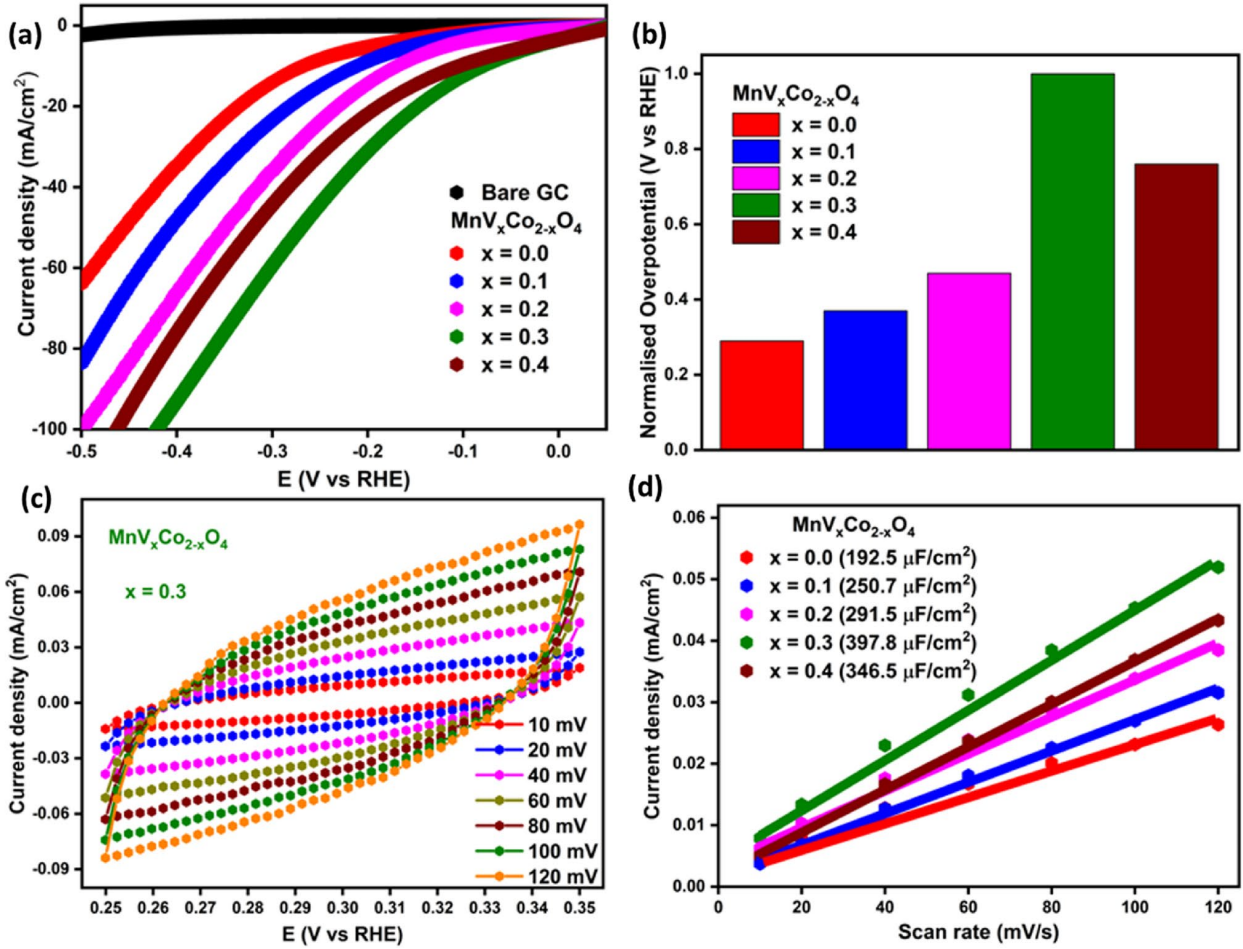
**(a)** LSV curves of V_x_-MnCo (x ≤ 0.4) MCs; **(b)** comparison plot between different catalysts of V_x_-MnCo MCs and their respective normalized overpotentials at the current density of 10 mA/cm^2^; **(c)** CV curves recorded in non-Ohmic region of −0.25 to −0.35 V versus RHE at different scan rates for V_x_-MnCo MCs (x = 0.3); **(d)** current density versus scan rate plot to estimate the value of C_dl_ for all the prepared samples.

The electrochemical performance of bare and various concentrations of V_x_-MnCo (x ≤ 0.4) MCs towards HER were assessed in 0.5 M H_2_SO_4_ electrolyte with a three-electrode configuration. The LSV measurements were carried out to investigate the electrocatalytic activity of representative electrodes. Figure 10a depicts the LSV acquired by scanning the samples within 0 to −0.5 V versus RHE at a 10 mV/s scan rate. It is worth mentioning that V_x_-MnCo (x = 0.3) MCs electrocatalyst has the lowest onset potential (−78.9 mV), making it the most efficient catalyst among V_x_-MnCo MCs. The designated V_x_-MnCo (x = 0.3) MCs exhibits remarkable HER activity in the overpotential of merely 85.9 mV to offer 10 mA cm^−2^ current density among other different composition of V_x_-MnCo MCs. These results revealed that V_x_-MnCo (x = 0.3) MCs performed very well as an HER electrocatalyst which could be attributed to the deprotonation of water molecules liberating a significant number of H species the V_x_-MnCo MCs readily absorb. Afterwards, it establishes chemical connections with nearby electrons or adsorbed hydrogen (H_ads_), and both of these processes coincide, resulting in the formation of H_2_ molecules. The remaining samples exhibited relatively low performance, possibly due to the fewer available active sites for the adsorption of hydrogen species and reduced electrochemical surface area. V doping is crucial to achieving enhanced HER because adding V increases the electrochemical surface area and results in many defective sites, which are advantageous for the rapid transfer of charges. Figure 10b illustrates a comparison plot of normalized overpotential values at 10 mA/cm^2^ for several samples. The lowest overpotential value of 85.9 mV exhibited by the V_x_-MnCo (x = 0.3) MCs electrocatalyst demonstrates the effectiveness of the composite in catalyzing HER (Table 2).

**Table 2:** HER parameters for different catalysts of V_x_-MnCo (x ≤ 0.4) MCs electrodes.

x	Onset potential (mV)	Overpotential (mV) at −10 mA/cm^2^	Tafel slope (mV/dec)	DLC (µF/cm^2^)	ESCA (cm^2^)
0.0	141.5	268.0	131	192.5	5.5
0.1	129.5	211.4	115	250.7	7.2
0.2	114.6	169.7	103	291.5	8.3
0.3	78.9	85.9	84	397.8	11.4
0.4	104.6	109.0	96	346.5	9.9

Electron spectroscopy for chemical analysis (ESCA) is a significant activity parameter relating to electrocatalytic performance, such as double layer capacitance (Cdl) and the specific capacitance (Cs)^46^. To confirm the assumption mentioned above for higher activity of V_x_-MnCo (x = 0.3) MCs, the ECSA was measured for bare and V_x_-MnCo MCs electrodes. The following Eq. (2) was utilized to calculate the ECSA.

2 $$\mathrm{ECSA }=\mathrm{ Cdl }/\mathrm{ Cs}$$

Cdl values were derived using the non-Faradaic potential region in CV curves, and Cs were assumed to be 35 µF cm^−2^ ^47^. Figure 10c depict the CV curves obtained for the V_x_-MnCo (x = 0.3) MCs electrocatalyst at scan speeds ranging from 10 to 120 mV/s in the potential range of −0.25 to −0.35 V versus RHE. Figure 10d illustrates the linear fit achieved by fitting the current density at a potential of −0.30 V versus RHE against several scan rates. The slope of these straight lines (Fig. 10d) was used to determine the value of C_dl_. The ESCA and C_dl_ values for all the prepared samples of V_x_-MnCo MCs have been displayed in Table 2. The electrocatalyst V_x_-MnCo (x = 0.3) MCs exhibited outstanding performance with an ECSA value of 11.4 cm^2^. To further examine the catalysts' number of active sites (N), the following Eq. (3) was utilized to calculate,

3 $$\mathrm{N }=\mathrm{ Qs }/\mathrm{ F}$$

where Qs is the surface charge density was derived by integrating the charge of each CV curve over the whole potential range, the half value of the charge was obtained as the Qs values, and F is the Faraday constant (96,485 C mol^−1^)^48^. According to the Qs values, the calculated number of active sites for all the prepared samples of V_x_-MnCo (x ≤ 0.4) MCs were 1.31 × 10^–7^, 1.35 × 10^–7^, 1.77 × 10^–7^, 2.41 × 10^–7^, and 1.82 × 10^–7^ mol cm^−2^, respectively. This result confirms that the electrocatalyst V_x_-MnCo (x = 0.3) MCs has more active sites for adsorbing the hydrogen molecules responsible for HER.

**Figure 11:**
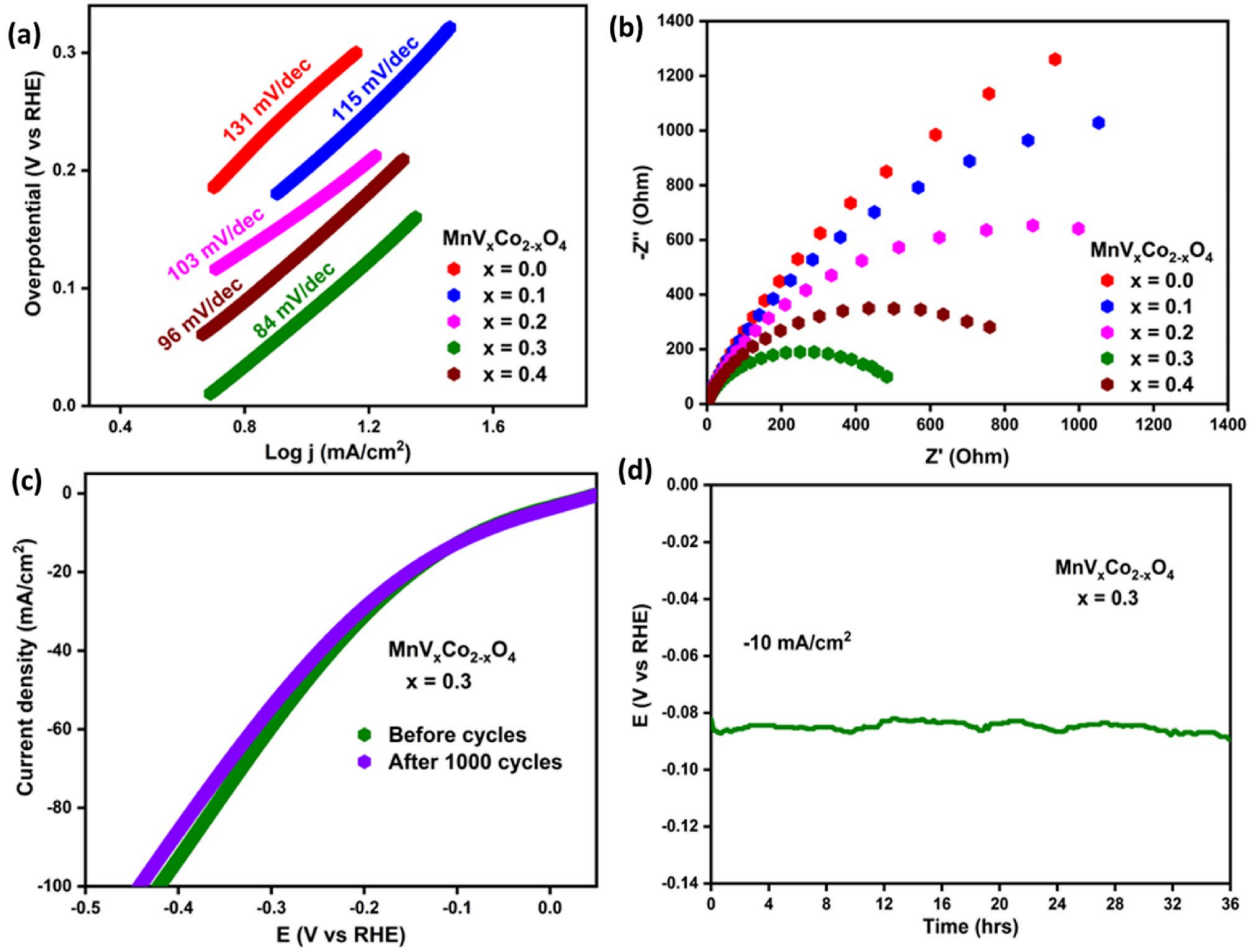
**(a)** Tafel plot for V_x_-MnCo (x ≤ 0.4) MCs; **(b)** Nyquist plot for all the prepared samples; **(c)** LSV curves of V_x_-MnCo (x = 0.3) MCs swept before and after 1000 CV cycles; **(d)** CP curve of V_x_-MnCo MCs (x = 0.3) MCs observed for a prolonged period of 36 h.

The kinetics of the electrocatalytic process was investigated using Tafel analysis, which relates the connection between potential and current density. The corresponding Tafel equation, as shown in Eq. (4)

4 $$\upeta =\mathrm{ a }+\mathrm{ b}\times \mathrm{log}(\mathrm{j})$$

where Tafel constant, a; the Tafel slope, b; the current density, j; overpotential, η^47^. Figure 11a illustrates Tafel plots for V_x_-MnCo MCs, with corresponding slopes of 131, 115, 103, 84, and 96 mV/dec, respectively. V_x_-MnCo MCs (x = 0.3) electrocatalyst exhibited Tafel slope values as low as 84 mV/dec, further supporting the assumption that this material adheres to the Volmer–Heyrovsky rate-determining step in HER. The values of the Tafel slopes represent the reaction route encountered during the catalytic performance. An initial stage also called the Volmer reaction, involves the hydrogen atom absorbing a discharged proton off the surface of the electrode. In the Heyrovsky process, H_ads_ will combine with a proton and an electron to produce new hydrogen molecules. The Tafel route suggests recombining two H_ads_ species yielding a hydrogen molecule. The Volmer–Heyrovsky pathway consists of the Volmer and Heyrovsky processes and is the most prevalent mechanism for the HER to proceed^48,49^. The calculated Tafel slope of 84 mV/dec for the most effective electrocatalyst V_x_-MnCo MCs (x = 0.3) signals that the reaction proceeds via the rate-determining Volmer–Heyrovsky step.

The intense electrocatalytic activity presented by the small semicircle generally shows fast electron transfer at the catalyst electrolyte interface. As illustrated in Fig. 11b, the EIS analysis was conducted to understand the catalyst's efficiency in transferring charges across the interface in 0.5 M H_2_SO_4_ electrolyte solution. The Nyquist plot of V_x_-MnCo MCs with an η of 85.9 mV and a frequency range of 0.1 Hz to 10^5^ Hz is depicted in Fig. 11b. V_x_-MnCo MCs (x = 0.3) electrocatalysts exhibited a lower charge transfer resistance than other catalysts, indicating that it significantly enhanced the HER performance, demonstrating a better ability to transfer electrons at the electrocatalysts/electrolyte interface.

**Figure 12:**
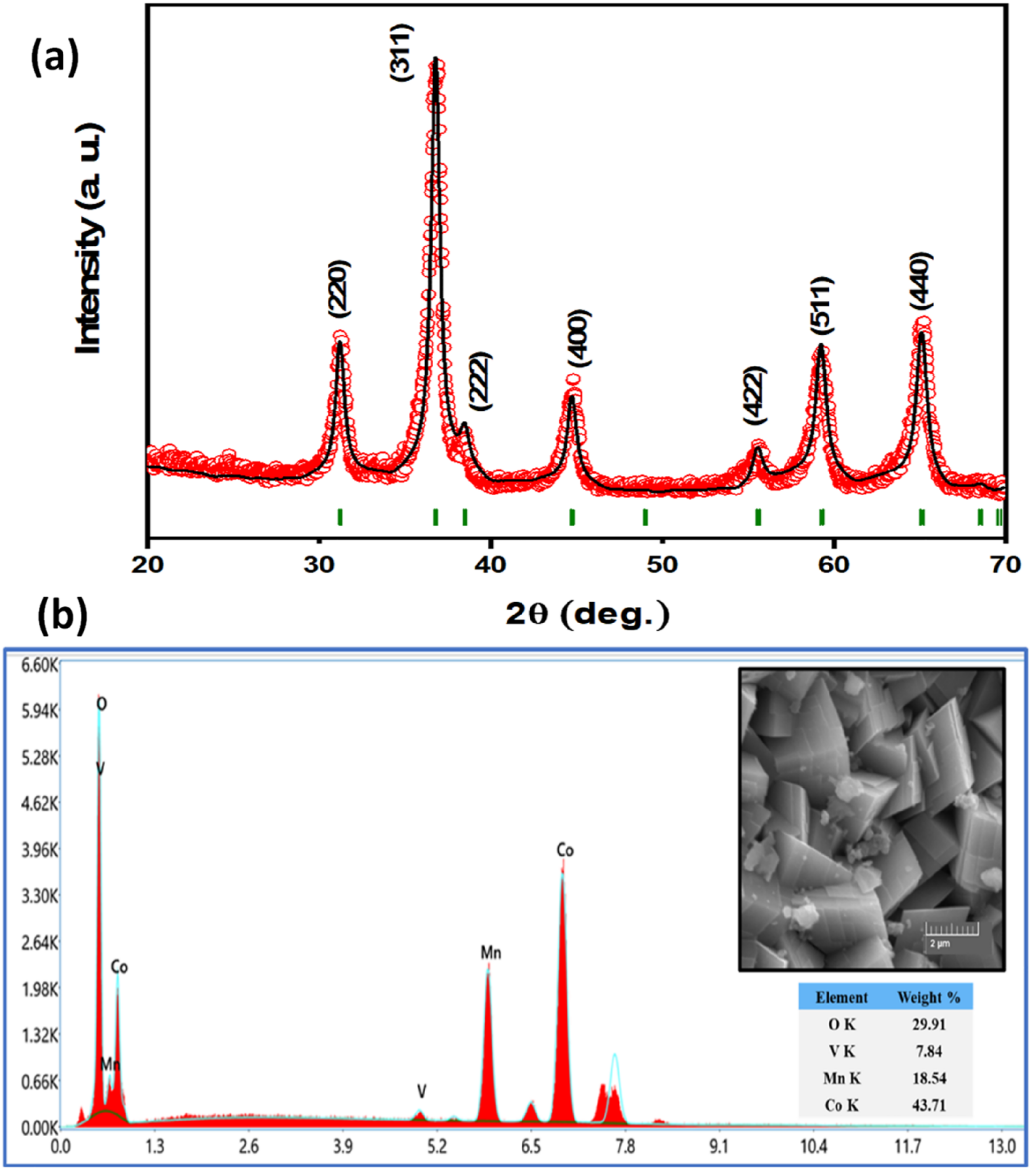
**(a)** XRD spectrum; **(b)** EDX spectrum and SEM image of V_x_-MnCo (x = 0.3) MCs after stability test.

Figure 11c depicts the LSV used to evaluate the stability of the V_x_-MnCo (x = 0.3) MCs electrocatalyst before and after CV analysis. As seen in Fig. 11c, after 1000 cycles of CV study, the LSV curve of V_x_-MnCo (x = 0.3) MCs electrocatalyst demonstrates almost a change of 4 mV in the overpotential, which may be due to the shredding of material from the electrode surface caused by the bubble formation. CP analysis was utilized to further confirm the long-term stability of the electrocatalyst by delivering a constant current of -10 mA/cm^2^ for 36 h in an acidic solution, illustrated in Fig. 11d. The little disruption observed in the CP curve of V_x_-MnCo (x = 0.3) MCs is due to the formation and concentration of bubbles during H_2_ release at the electrode surface. However, the electrocatalyst exhibited excellent potential value retention even after 36 h of electrocatalysis at a current density of −10 mA/cm^2^. Following the stability tests, the structure, morphology, and composition of the V_x_-MnCo (x = 0.3) MCs were re-evaluated (Fig. 12). However, the morphology was exhibited chunky of highly agglomerated cubic particles.

Furthermore, no significant changes in the structure and composition were observed. This provides conclusive evidence that V_x_-MnCo (x = 0.3) MCs electrocatalysts are highly stable. These findings demonstrate that V_x_-MnCo (x = 0.3) MCs is a high-performance HER electrocatalyst suitable for future hydrogen fuel cell technology. The overpotential comparison of electrocatalytic HER performance of V_x_-MnCo (x = 0.3) MCs with other HER catalysts reported in the literature is listed in Table 3.

**Table 3:** The overpotential comparison of electrocatalytic HER performance of MnV_x_Co_2−x_O_4_ with other HER catalysts in an acidic (0.5 M H_2_SO_4_) medium was reported in literatures.

Catalysts	Overpotential, η (mV) at 10 mA/cm^2^	References
MnV_x_Co_2−x_O_4_	85.9	This work
Mn_0.9_Co_0.1_Al_2_O_4_	110	^50^
NC/CuCo/CuCoO_x_	112	^51^
CoSe_2_/carbon fiber	139	^52^
NiFe_2_O_4_	150	^53^
MnCo_2_O_4_(50%)/Ti_3_C_2_T_x_	177	^54^
Exf-NiFe_2_O_4_/CB	187	^55^
NiCo_2_O_4_ nanowires	190	^56^
FeSe_2_/CoFe_2_O_4_	231	^57^
CoFe_2_O_4_-graphene	248.3	^58^
NiFe_2_O_4_-graphene	259	^58^
MnFe_2_O_4_-graphene	313	^59^
ZnFe_2_O_4_-graphene	315	^59^
Porous NiCo_2_O_4_	370	^60^
Ce-MnCo_2_O_4_	389	^13^
SrLaAl_1/2_Mn_1/2_O_4_	453	^61^
Sr_2_LaCoMnO_7_	612	^62^

## Conclusion

In summary, V_x_-MnCo (x ≤ 0.4) MCs were synthesized effectively using a simple hydrothermal process. These sample' structure, morphology, magnetic properties, and their possible usage as efficient HER electrocatalysts were investigated. Both structural and morphological analyses done by XRD, HR-TEM and TEM confirmed the formation V_x_-MnCo (x ≤ 0.4) MCs with minor impurity of MnO_2_. The different products display a paramagnetic behaviour at higher temperatures and a ferrimagnetic behaviour at lower temperatures. It is detected that the Curie temperature (T_c_) and peak temperature in M_ZFC_ curves increase with the rise in vanadium concentration. From both M-H and M-T results, an increase in the global magnitude of magnetization is detected with decreasing the temperature, which is mainly correlated with the reduction in thermal agitation. M-H results show that M_max_ (± 70 kOe) values at both 300 and 10 K and M_r_ value increase as the vanadium content increases. The Hc value increases with increasing "x" up to 0.2, then drops with a further rise in “x” above 0.2. The V_x_-MnCo (x = 0.3) MCs electrode has outstood HER activity much better than its other counterparts. The optimized catalyst V_x_-MnCo (x = 0.3) MCs electrode requires a low onset potential of 78.9 mV, low overpotential of 85.9 mV at 10 mA/cm^2^, and a slight Tafel slope of 84 mV/dec in 0.5 M H_2_SO_4_ electrolyte. The excellent performance of V_x_-MnCo (x = 0.3) MCs is due to the increased ECSA and electron transport capacity at the electrode/electrolyte interface.

These results revealed that Vx-MnCo (x = 0.3) MCs are a promising candidate electrode for the electrochemical HER in large-scale commercial hydrogen fuel cell applications. Further, long-term testing has shown that this electrode keeps high quality even after 1,000 CV cycles and 36 h of CP testing. Also, with the magnetic property advantage, the magnetic field-induced electrochemical catalytic property of these catalysts may provide a new perspective for the field of magnetoelectrochemistry in future.

## Data Availability

The datasets used and/or analyzed during the current study are available from the corresponding author on reasonable request.
